# Development of Parallel Reaction Monitoring Mass Spectrometry Assay for the Detection of Human Norovirus Major Capsid Protein

**DOI:** 10.3390/v14071416

**Published:** 2022-06-28

**Authors:** Yayoi Kimura, Jihye Shin, Yusuke Nakai, Masaya Takahashi, Yoko Ino, Tomoko Akiyama, Keiko Goto, Noriko Nagata, Yutaro Yamaoka, Kei Miyakawa, Hirokazu Kimura, Akihide Ryo

**Affiliations:** 1 Advanced Medical Research Center, Yokohama City University, Yokohama 236-0004, Japan; ykimura@yokohama-cu.ac.jp (Y.K.); jihye@yokohama-cu.ac.jp (J.S.); yusuke06@yokohama-cu.ac.jp (Y.N.); t216509g@yokohama-cu.ac.jp (M.T.); yino@yokohama-cu.ac.jp (Y.I.); akiyama@yokohama-cu.ac.jp (T.A.); 2 Department of Microbiology, School of Medicine, Yokohama City University, Yokohama 236-0004, Japan; yamaoka.yut.vs@yokohama-cu.ac.jp (Y.Y.); keim@yokohama-cu.ac.jp (K.M.); 3 Department of Virology, Ibaraki Prefectural Institute of Public Health, Mito 310-0852, Japan; t176023b@yokohama-cu.ac.jp (K.G.); n.nagata@pref.ibaraki.lg.jp (N.N.); 4 Life Science Laboratory, Technology and Development Division, Kanto Chemical Co., Inc., Isehara-shi 259-1146, Japan; 5 Department of Health Science, Gunma Paz University Graduate School, Takasaki-shi 370-0006, Japan; h-kimura@paz.ac.jp

**Keywords:** Norwalk virus, norovirus, stool specimen, mass spectrometry, parallel reaction monitoring (PRM)

## Abstract

Human Norwalk viruses (HuNoVs), the most common etiological agents of acute gastroenteritis, are genetically diverse RNA viruses that frequently cause mass food poisoning internationally. Although nucleic acid detection methods, such as reverse transcription–quantitative polymerase chain reaction (RT-qPCR), are the gold standard for the diagnosis of norovirus infection, alternative methods are needed for the specific and sensitive viral protein detection for rapid diagnosis and surveillance. In this study, we developed a robust and high-throughput targeted proteomic assay workflow to directly detect the VP1 major capsid protein of HuNoVs. A parallel reaction monitoring (PRM) assay using a high-resolution mass spectrometer was used to detect representative peptides derived from VP1 in six different HuNoV genotypes. An optimized protocol using synthesized heavy isotope-labeled peptides as internal standards was also used to simultaneously genotype and quantify the VP1 protein in human stool specimens. This method is expected to become a new tool for studying the molecular epidemiology of HuNoV and to shed new light on targeted proteomics in clinical practice.

## 1. Introduction

Norwalk viruses (NoVs) are the primary viruses identified as causing acute viral gastroenteritis in mammalian hosts, and they are now recognized as an important cause of epidemics of sporadic gastroenteritis in both children and adults [1]. The human Norwalk virus (HuNoV) spreads quickly in crowded environments as a consequence of its low infectious dose (<10^2^ viral particles), prolonged asymptomatic shedding, high environmental stability, and broad strain diversity [2]. Therefore, controlling HuNoV endemics and outbreaks poses a major challenge for sustainable public health and disease prevention.

The RNA genome of HuNoV consists of three open reading frames (ORF 1–3) [3,4]. Among them, ORF2 encodes the basic building unit of viral capsid protein VP1 (58–60 kDa). On the basis of recent phylogenetic analyses of the VP1 gene and partial RNA-dependent RNA polymerase gene, NoVs are classified into 10 genogroups (GI–GX) and 49 genotypes [5]. Most HuNoVs belong to genogroups GI, GII, or GIV. Because VP1 is the most abundant protein constituting the major component of capsids in viral particles and is responsible for viral infectivity during an outbreak, VP1 is a potential target for the diagnosis of HuNoV infections [6].

HuNoV has been diagnosed by electron microscopy (EM) [7,8]. However, the sensitivity of EM detection is low, with a detection limit of 10^6^ viral particles per gram of feces, and it requires a skilled operator and sophisticated setup for continuous implementation. Reverse transcription–quantitative polymerase chain reaction (RT-qPCR) is currently the most common technique for genotyping HuNoV in stool specimens, water, and food [8,9,10,11,12]. However, this method detects only viral mRNA and sometimes might not reflect protein expression. In addition, it requires not only the use of multiple PCR primers for various HuNoV genotypes but also a certain turnaround time. Multiplex PCR, which involves genotyping from a single sample in a single reaction, is often more complex to develop and less sensitive than PCR with a single primer set. Other studies have used methods based on immunological applications such as lateral flow immunoassays (LFIAs) [13] and enzyme-linked immunosorbent assays (ELISAs) [11,12,14]). Many of these methods are semi-quantitative and moderately sensitive and use antibodies that are insufficient to distinguish between different genotypes. Therefore, a compelling need exists to develop rapid and highly sensitive diagnostic and genotyping assays that directly detect the HuNoV antigen in patient specimens.

The parallel reaction monitoring (PRM) assay is a powerful, mass spectrometry (MS)-based targeting approach that enables the detection and quantification of pre-specified target peptides with high resolution, sensitivity, and throughput [15,16]. The targeted MS approach can also potentially identify different genotypes in complex samples and, once established, can be implemented relatively quickly in various contexts. In addition, absolute quantification can be achieved using heavy isotope-labeled peptides as internal standards.

In this study, we established targeted proteomics for the detection and quantitation of HuNoV capsid protein VP1. Recombinant full-length VP1 proteins from six different genotypes were synthesized and then subsequently analyzed by tryptic digestion and data-dependent acquisition mass spectrometry (DDA-MS) to select target peptides for PRM assays. Consequently, the PRM assay using synthesized heavy isotope-labeled peptides could successfully detect NoV VP1 proteins in human stool specimens of patients with norovirus infection. The PRM assay was also able to quantify the amounts of viral antigens and simultaneously determine the virus genotypes.

## 2. Materials and Method

### 2.1. HuNoV VP1 Protein Synthesis and Sample Preparation for MS Analysis

Complementary DNAs encoding VP1 of HuNoV GI.1 (M87661), GII.2 (X81879), GII.3 (U02030), GII.4 (X76716), GII.5 (AJ277607), GII.6 (AJ277620), and GII.17 (AY502009) were chemically synthesized. Synthetic cDNAs were inserted into the pEU-bls-s1-MCS vector using an In-Fusion HD Cloning kit (Takara Bio, Kusatsu, Japan). Seven genotypes of biotinylated HuNoV VP1 protein were produced by in vitro transcription and cell-free protein synthesis using wheat germ extract (cat. WEPRO7240H; CellFree Sciences, Yokohama, Japan) in the presence of biotin ligase and 0.5 µM of d-biotin, as previously described [17,18]. For the MS analysis, each recombinant protein was isolated from the cell-free translation reaction mixture (100 µL) using Streptavidin Sepharose High Performance (5 µL; GE Healthcare, Piscataway, NJ, USA) and then transferred to a 0.22 µm centrifugal filter unit (Merck, Darmstadt, Germany). After being washed twice with 50 mM Tris-HCl (pH 7.5) containing 0.5 M NaCl and 0.2% sodium dodecyl sulfate (SDS), the resins were washed twice with phosphate-buffered saline (PBS) and three times with serially distilled water. All water-soluble materials were removed by centrifugation at 10,000× *g* at room temperature for 1 min. For on-bead tryptic digestion, the resin was resuspended in 40 µL of 50 mM NH_4_HCO_3_ containing 2 M urea, and proteins were subsequently reduced with dithiothreitol (final concentration of 10 mM) at 37 °C for 15 min, and alkylated with 2-iodoacetamide (final concentration of 25 mM) at room temperature for 15 min. Tryptic digestion with Trypsin Gold (Promega, Madison, WI, USA) was performed overnight at 37 °C. After centrifugation, the resultant peptides were desalted using a StageTip [19], and the subsequently eluted peptides were completely lyophilized and maintained at −30 °C until use. Peptide mixture quantification was performed using a NanoDrop ND-1000 (Thermo Fisher Scientific, Waltham, MA, USA).

### 2.2. Human Stool Specimen Preparation for MS Analysis

All human stool samples were obtained from patients with norovirus infection and stored at −80 °C until use. The determination of HuNoV genotype was performed using RT-qPCR [20]. For the protein extraction from human stool specimens, 100–200 µL of frozen stool samples were thawed on ice and diluted in 10 mL of PBS (pH 7.4), vortexed thoroughly to disrupt, and then centrifuged at 3000× *g* for 10 min. The supernatant was collected and filtrated using a 0.22 μm syringe filter (Merck) and then subjected to trichloroacetic acid precipitation. After being washed with 80% acetone, all the samples were denatured by adding 50 mM Tris-HCl (pH 8.2) containing 8 M urea and 75 mM NaCl. Total protein concentrations from each sample were determined by a Bradford assay using the Bio-Rad Protein Assay kit (Bio-Rad, Hercules, CA, USA). SDS–polyacrylamide gel electrophoresis was performed using a SuperSep Ace 5–20% gradient gel (FUJIFILM Wako Pure Chemical, Osaka, Japan), followed by detection with SYPRO Ruby staining (Lonza, Rockland, ME, USA). For MS analysis, proteins (20 μg) were reduced with dithiothreitol (final concentration of 10 mM) and alkylated with 2-iodoacetamide (final concentration of 25 mM). The protein solutions were diluted from 8 M to 2 M urea in 50 mM NH_4_HCO_3_ and then incubated with Trypsin Gold at 37 °C overnight. To prepare the resultant peptides for MS analysis, the solutions were desalted using a StageTip [19]. The subsequently eluted peptides were completely lyophilized and then stored at −30 °C until use.

### 2.3. Liquid Chromatography–Tandem Mass Spectrometry Analysis

Desalted peptides (L) and the heavy isotope-labeled peptides synthesized with C-terminal [^13^C^6^, ^15^N^2^] lysine and [^13^C^6^, ^15^N^4^] arginine (Scrum, Tokyo, Japan) (H) were resuspended in 0.1% formic acid and 2% acetonitrile (ACN). All MS analyses were carried out using a Q-Exactive HF mass spectrometer (Thermo Fisher Scientific) coupled with an UltiMate 3000 high-performance liquid chromatography (HPLC) system (Thermo Fisher Scientific). MS data were acquired using either DDA or PRM.

### 2.4. Data Analysis

To identify peptides, DDA-MS data were analyzed using the SEQUEST HT search algorithm in Proteome Discoverer (version 2.2; Thermo Fisher Scientific) against in silico translations from the VP1 nucleotide sequence database, allowing for methionine oxidation as a variable modification and cysteine carbamidomethylation as a fixed modification. Other parameters used for the search were as follows: Trypsin digestion with two missed cleavages permitted, 300–6000 Da, 6 minimum length of peptide, 40 maximum length of peptide, peptide mass tolerance for MS data ± 10 ppm, and fragment mass tolerance ± 0.05 Da. We used a 1% stringent false discovery rate as a cutoff value for peptide identification. The Skyline software (version 20.1.0.31; MacCoss Lab Software, University of Washington, WA, USA) [21] was used to create the VP1 spectral library for the PRM assay from Proteome Discoverer search file datasets and to perform peak integration and quantification of the targeted VP1 proteins from PRM raw files. All peaks were manually inspected to ensure that precursor and fragment ions were detected correctly. Reverse calibration curves were generated for each peptide pair by spiking heavy isotope-labeled peptides at concentrations from 0.191 fmol to 100 pmol in 100 ng of a recombinant HuNoV VP1 protein mixture as a light peptide and 100 ng of HeLa cell lysate as the background. To improve the detection accuracy of the target peptides by the PRM method, the identification criteria were as follows: At least four fragment peaks were detected, the retention time and transition ratio of the heavy peptides (H) and light peptides (L) were the same, and the mass deviation between them was less than 5 ppm. The total area fragments of the target peptide were calculated as the sum of the peak areas of four fragment ions (rank 1–4) for each of the target peptides. The spectral peak areas of the target peptides were manually calculated by integrating the fragment peak area.

## 3. Results

### 3.1. Determination of PRM Analysis Target Peptides Using Recombinant Human Norovirus VP1

**Figure 1:**
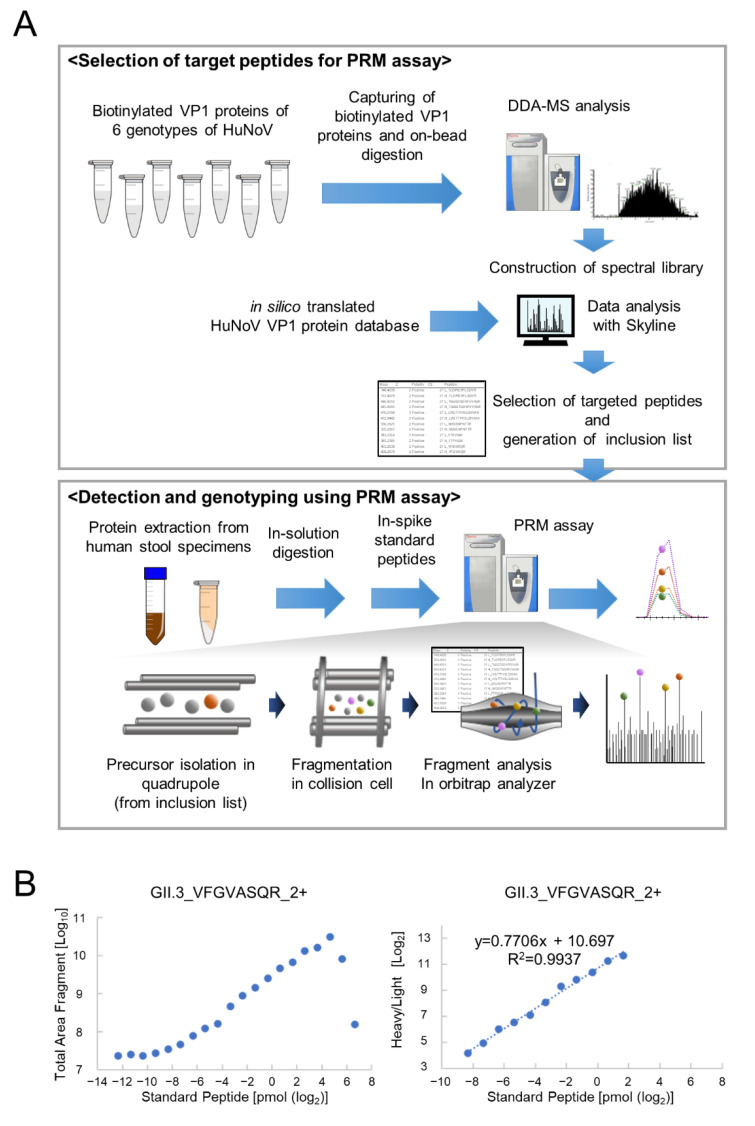
PRM assay construction for detection and genotyping of the virus. (**A**) Experimental workflows for PRM assay construction for HuNoV VP1 detection. The peptide information of recombinant HuNoV VP1 proteins was obtained through DDA-MS analysis and in silico translation from the VP1 nucleotide sequence database. The retention time of each heavy isotope-labeled synthetic peptide and the fragment ions’ rank in the PRM assay were analyzed using the Skyline software. Target peptides from the inclusion list were isolated and fragmented from the sample on the basis of the mass-to-charge ratio, and all fragments were analyzed in parallel on a high-resolution mass spectrometer. (**B**) Example of a calibration curve. The calibration curve was created using the total area fragment of peptide ions. The horizontal axis represents the quantity of standard peptides injected; the vertical axis represents the total area fragment of heavy isotope-labeled peptide ion (left), and the light/heavy ratio (right) obtained using the PRM assay.

For construction of the PRM assay for HuNoV VP1 detection, we created the VP1 spectral library and selected target peptides (Figure 1A). In the present study, seven genotypes of biotinylated recombinant full-length HuNoV VP1 proteins were first produced using the wheat germ cell–free protein synthesis system (Supplementary Figure S1), followed by on-bead tryptic digestion. DDA-MS analysis detected 12–43 peptides from each genotype of HuNoV VP1 with 16–51% coverage (Supplementary Table S1). Among these peptides, VP1 protein target peptides were selected for PRM assays using the Skyline software under the following conditions: Unique peptides from each genotype, 6 ≤ amino acid length ≤ 25, 300–1300 *m*/*z* (the mass-to-charge ratio), no cleavage misses, and double- or triple-charged precursor ions. In addition, peptides with C-terminal ragged ends (-KR, -RK, -RR, -KK, etc.), peptides with proline at P2 (xxxxPK/R) or P3 (xxxxPxK/R), peptides with methionine, and peptides with N-terminal glutamine were excluded from the candidate target peptides for quantitative analysis by the PRM assay (Supplementary Table S1). After selecting two or three more suitable peptides for each genotype of HuNoV VP1, we ultimately determined 15 targets from six genotypes of NoV VP1 and then synthesized these heavy isotope-labeled peptides with C-terminal [^13^C^6^, ^15^N^2^] lysine and [^13^C^6^, ^15^N^4^] arginine as internal standards (Table 1). In the GII.5 HuNoV VP1 protein, no unique peptide suitable for genotyping was found.

### 3.2. Optimization of PRM Assay Workflow and Quantification of VP1 Protein

To determine the lower limit of detection (LLOD) and the lower limit of quantification (LLOQ) in the PRM assay, reverse calibration curves were created by spiking heavy isotope-labeled peptides onto peptides derived from HeLa cell lysate and seven genotypes of recombinant HuNoV VP1 proteins. The linearity range of the calibration curve was defined as the range in which the coefficient of variation (CV) of the concentration of the recombinant VP1 protein (light peptide) was within 25% (Table 1). Figure 1B represents the results of peptide VFGVASQR (2^+^) from GII.3 as an example calibration curve. For VFGVASQR (2^+^), the LLOD was less than 0.1 fmol, the LLOQ was determined to be ~3 fmol, and linearity was excellent. Calibration curves for the other peptides are shown in Supplementary Figure S2, and the determined LOD, LLOQ, and upper limit of quantification (ULOQ) for 12 peptides from the six genotypes are shown in Table 1. Ultimately, a calibration curve with four standard peptides could not be developed in this study. As a result, 12 target peptides were selected from six genotypes of the HuNoV VP1 protein, enabling genotyping and quantitative monitoring using a PRM assay with sufficient sensitivity and reproducibility even for complex samples.

### 3.3. Quantification of HuNoV VP1 by the Developed PRM Assays in Individual Human Stool Specimens

**Figure 2:**
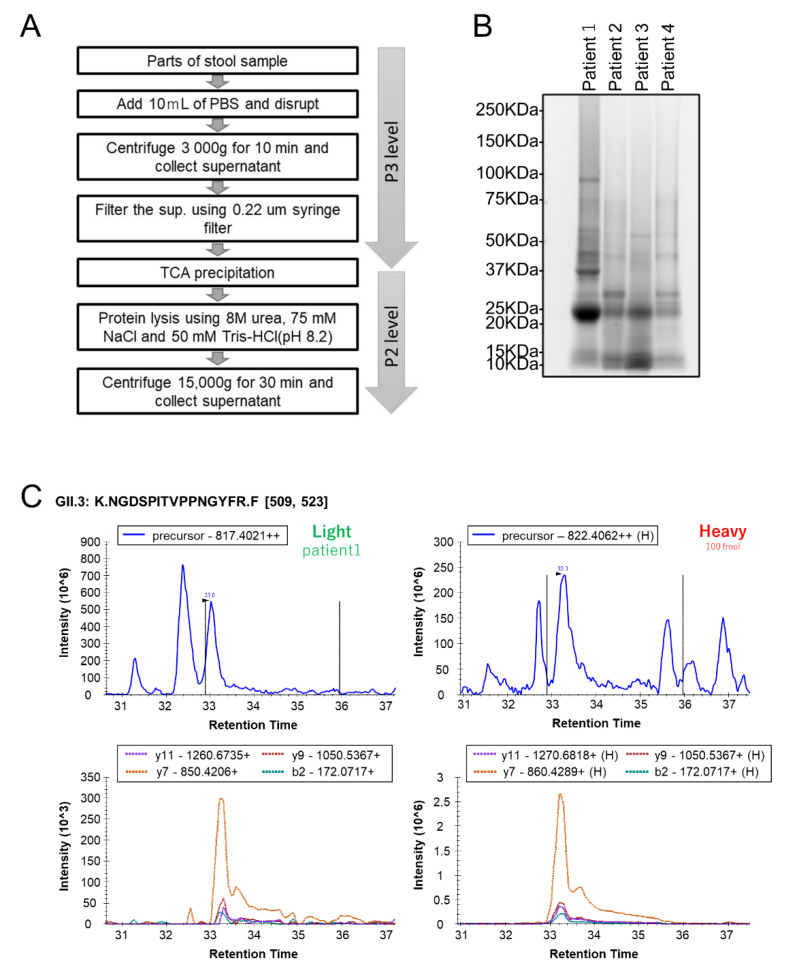
Targeted proteomic analysis of clinical stool specimens using the PRM assay. (**A**) Experimental workflows for protein extraction from a human stool sample. (**B**) The whole protein lysates obtained from clinical samples were separated using a 5–20% polyacrylamide gel, followed by detection using SYPRO Ruby staining. Each list of proteins detected by shotgun DDA-MS analysis is shown in Supplemental Table S2. Lane 1, Patient 1; Lane 2, Patient 2; Lane 3, Patient 3; Lane 4, Patient 4. (**C**) Example of PRM assay results for Patient 1 infected with the GII.3 VP1 genotypes of HuNoV.

Human stool specimens are noninvasive and readily accessible samples rich in viruses in patients with infectious diseases. Therefore, to evaluate the quality of the PRM assays for detecting VP1 proteins, we performed targeted proteome analysis using stool specimens from patients with acute gastroenteritis infected with HuNoV. In this study, to reduce the host/microbial proteins ratio, we used PBS suspended matter from stool specimens for MS analysis (Figure 2A). As a result, proteins (101–693 μg) were extracted from stool specimens of four patients infected with HuNoV whose VP1 genotypes determined by RT-qPCR were GII.2, GII.3, or GII.4 (Table 2 and Figure 2B). After tryptic digestion, the obtained peptides spiked with heavy isotope-labeled peptides (100 fmol of each peptide) as internal standards were analyzed by PRM assay. This whole procedure required approximately 8.5 h, which is only slightly longer than the corresponding assay by RT-qPCR. The PRM assay detected the VP1 target peptide of each genotype in all the stool specimens. The genotypes identified were consistent with the results of PCR analyses (Table 2). Our results suggest that the PRM assay, in conjunction with a high-resolution mass spectrometer, can be used to detect HuNoV and to furthermore identify genotypic HuNoV by detection of unique target peptides for VP1. In addition, the HuNoV VP1 peptides identified from each sample were quantitatively analyzed using each corresponding heavy isotope-labeled peptide as an internal standard. The concentrations of some target peptides obtained from stool samples were not determined because their concentrations were below the LLOQ in the calibration curve for each peptide (Table 1 and Table 2).

## 4. Discussion

The development of rapid and accurate detection techniques will help prevent the spread of viruses and initiate optimal therapy. Innovations in nucleic acid analysis technology, such as RT-qPCR, and the development of methods based on immunological applications, such as ELISA, have profoundly changed viral diagnosis [8,11,12,20]. Similarly, MS-based proteomic methods are expected to contribute significantly to improving the diagnosis of complex viral infections. Nevertheless, MS-based analysis is not yet routinely used to detect viruses in biological samples. One reason for the slow implementation of MS-based analysis might be that, unlike nucleic acids, proteins do not have an amplification method, making the development of a highly sensitive assay system difficult. However, targeted proteomic assays certainly have some advantages [15,16]. Given the genetic and antigenic diversity of HuNoVs, RT-qPCR and immunoassays require multiple primers and antibodies, respectively, to distinguish between different genotypes. In particular, immunoassays are not suitable for genotype assignment. In comparison, the targeted proteomic analysis, which measures only specific peptides by MS analysis, can analyze multiple targets, enabling genotyping of various viruses in a single analysis. In the present study, using 12 unique target peptides, we developed a PRM-based proteomics assay to detect and concurrently identify six different genotypes of the NoVs in a single MS analysis. Consequently, HuNoV VP1s in human stool specimens were successfully detected and genotyped. This study might provide useful information for the construction of a new assay system to identify viruses. To better identify the types and variants of various viruses using the PRM assay, it is necessary to determine unique target peptides specific to each virus. However, the assay can be easily extended to multiplex panels for multiple viruses, which compensates for the limitation of current diagnostic methods and is expected to be a future virus diagnostic tool. In the future, this technology could therefore be used to provide a comprehensive diagnosis of multiple viral infections caused by other enteric viruses in a single analysis for a single sample.

For most MS-based analyses, the typical dynamic range of detection is four to six orders of magnitude from high to low concentrations [22]. Therefore, the detection of low-abundance proteins in biological samples poses a challenge without pre-fractionation or immunodepletion steps. In stool specimens analyzed by MS, proteins of host or microbiota origin affect the identification of viral proteins. In this study, we therefore collected PBS-suspended matter from stool specimens of patients infected with HuNoV, resulting in reduced complexity and increased detection efficiency. However, DDA-MS analysis of the same samples revealed the proteome derived from the host and microbiota. More than 300 proteins of the human and bacterial flora in stool were detected (Supplementary Table S2). These findings may provide useful information for understanding the intestinal environment of patients with acute viral gastroenteritis. The proteomic analysis by MS does not require amplification or culture of the viral genome and thus can directly reflect the clinical reality of the patient with a viral infection. Hence, proteomic analysis of stool might be a powerful tool for improving treatment algorithms for acute gastroenteritis as well as viral diagnosis.

This study has limitations in showing that our PRM assay can be used as a routine diagnostic test for NoVs in acute gastroenteritis. One of these limitations is a lack of validation using multiple clinical samples of virus–positive and healthy individuals. Further studies with larger patient sample numbers as compared with healthy control subjects are needed to assess the quality of the PRM assays for detecting virus proteins. Further optimization of protein preparation methods from stool specimens is also needed to improve the detection sensitivity and reduce the processing time. In addition, this diagnostic method requires a highly sensitive and well-maintained mass spectrometer that can handle extremely low amounts of viral protein expression.

## 5. Conclusions

In this study, we constructed a HuNoV detection system utilizing a PRM assay in conjunction with a high-resolution mass spectrometer. The PRM assay could identify genotypic HuNoV because it could detect unique target peptides for VP1 in some genotypes. Further technological developments are required before this technique can be used to comprehensively diagnose multiple viral infections and improve therapeutic algorithms for acute gastroenteritis.

## Figures and Tables

**Table 1:** Target peptides for a PRM assay of HuNoV VP1 protein.

Norovirus VP1	Peptide	Start-End	Charge	Light *m*/*z*	Heavy *m*/*z*	Fragment Ions Analyzed	LLOD * (fmol)	LLOQ ** (fmol)	ULOQ *** (pmol)	CV of Light Peptide in the Linearity Range (%)
GI.1_M87661/ Norwalk/68/US	TLDPIEVPLEDVR	154–166	2	748.404	753.408	y10^+^, y8^+^, y6^+^, y2^+^	<0.19	780	>100	23.19
	TGGGTGDSFVVAGR	192–205	2	640.815	645.819	y9^+^, y7^+^, y4^+^, y3^+^	<0.19	3.05	25	23.92
	LVGTTPVSLSHVAK	276–289	3	470.277	472.948	y9^+^, y8^+^, y7^+^, y2^+^	<0.19	6.1	12.5	23.09
GII.2_X81879/ Melksham	VFGVISQR	332–339	2	453.264	458.268	y6^+^, y4^+^, y3^+^, b3^+^	<0.19	3.05	12.5	23.10
	YAGALNLNTNLAPSVAPVFPGER	410–432	2	1186.124	1191.128	y12^+^, y11^+^, y7^+^, y4^+^	<0.19	780	12.5	6.97
GII.3_U02030/ Tronto	VFGVASQR	343–340	2	432.240	437.244	y6^+^, y4^+^, b2^+^, b3^+^	<0.19	3.05	3.13	23.15
	SQLPSSGGR	443–451	2	444.730	449.734	y7^+^, y6^+^, y2^+^, b2^+^	N.D.	N.D.	N.D.	N.D.
	NGDSPITVPPNGYFR	510–524	2	817.402	822.406	y11^+^, y9^+^, y7^+^, b2^+^	<0.19	1.53	50	19.38
GII.4_X76716/ Bristol	NNFYHYNQANDSTLK	163–177	3	610.280	612.951	y7^+^, y6^+^, y4^+^, b2^+^	<0.19	6.1	6.25	23.05
	ANNAGDDVFTVSCR	188–201	2	763.339	768.343	y10^+^, y6^+^, y3^+^, b2^+^	<0.19	6.1	>100	21.92
GII.6_AJ277620/ Seacroft/90/UK	GTLISQTAR	287–295	2	473.769	478.774	y7^+^, y6^+^, y5^+^, y1^+^	<0.19	1.53	6.25	15.95
	NHPLHVQVK	307–315	3	357.873	360.545	y5^+^, y4^+^, y1^+^, b2^+^	N.D.	N.D.	N.D.	N.D.
	LGTILIK	380–386	2	379.263	383.270	y2^+^, y1^+^, b3^+^, b2^2+^	<0.19	3.05	0.39	19.53
GII.17_AY502009/ CS-E1/2002/US	NVGSNPNTTR	340–349	2	530.263	535.267	y8^+^, y5^+^, y1^+^, b2^+^	N.D.	N.D.	N.D.	N.D.
	FTPVGIK	387–393	2	381.231	385.239	y5^+^, y3^+^, y1^+^, b2^+^	<0.19	6.1	12.5	19.65

*, Lower limit of detection; **, Lower limit of quantification; ***, Upper limit of quantification; N.D., not determined.

**Table 2:** Detection of VP1 genotype in human stool specimens using the PRM assay.

Patient No.	PCR Analysis		Extracted Total Protein (μg)	PRM Assay	
	Genotype	Ct Value		Detected Target Peptide	Concentration (fmol/μg)
1	GII.3	19	693	GII.3 NGDSPITVPPNGYFR_2+	13.17
				GII.3 VFGVASQR_2+	<LLOQ *
2	GII.4	19	319	GII.4 ANNAGDDVFTVSCR_2+	<LLOQ *
3	GII.P16/GII.2	18	545	GII.2 YAGALNLNTNLAPSVAPVFPGER_2+	5.99
4	GII.P16/GII.2	19	101	GII.2 YAGALNLNTNLAPSVAPVFPGER_2+	<LLOQ *

*, Less than the lower limit of quantification.

## Data Availability

All mass spectrometry proteomics data have been deposited to the ProteomeXchange Consortium (http://www.proteomexchange.org) via the jPOST (https://jpostdb.org) partner repository with the dataset identifier PXD033981. All data are fully available without restriction.

## Supplementary Materials

The following supporting information can be downloaded at: https://www.mdpi.com/article/10.3390/v14071416/s1, Figure S1: Synthesis of seven genotypes of biotinylated recombinant full-length HuNoV VP1. Figure S2: Calibration curve of peptides derived from VP1 proteins. Table S1: The peptide list from human HuVP1 recombinant proteins detected by DDA-MS analysis. Table S2: The protein list from human proteins detected by DDA-MS analysis.
