# Supplementary material for: Development of Parallel Reaction Monitoring Mass Spectrometry Assay for the Detection of Human Norovirus Major Capsid Protein
Source: Viruses. 2022 Jun 28;14(7):1416. doi: 10.3390/v14071416 (PMC9319599; doi:10.3390/v14071416)
Supplement: Supplementary file 1 [file viruses-14-01416-s001.zip › Supplementary Figure.pptx]

## Slide 1
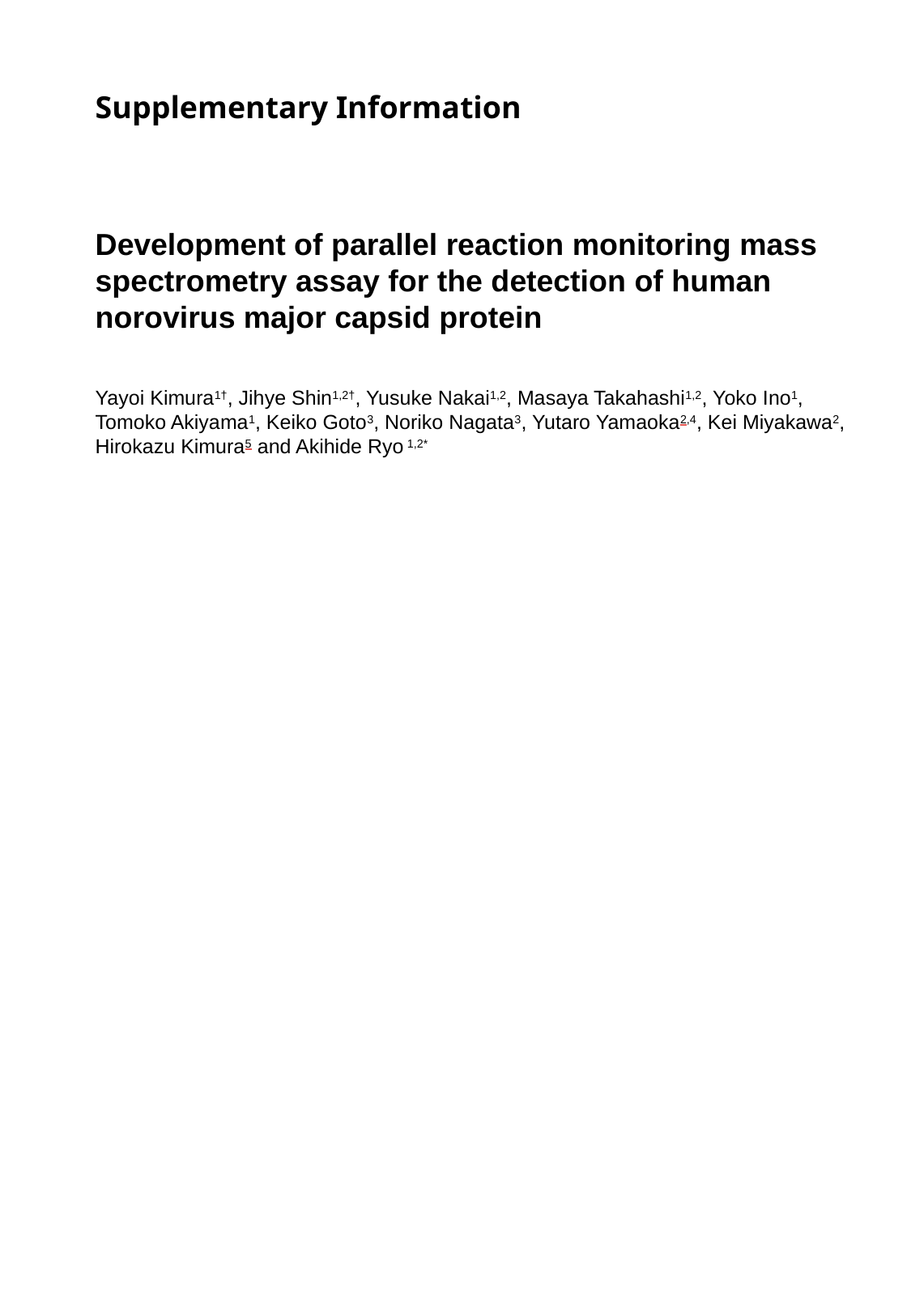

Supplementary Information
Development of parallel reaction monitoring mass spectrometry assay for the detection of human norovirus major capsid protein
Yayoi Kimura1†, Jihye Shin1,2†, Yusuke Nakai1,2, Masaya Takahashi1,2, Yoko Ino1, Tomoko Akiyama1, Keiko Goto3, Noriko Nagata3, Yutaro Yamaoka2,4, Kei Miyakawa2, Hirokazu Kimura5 and Akihide Ryo 1,2*

## Slide 2
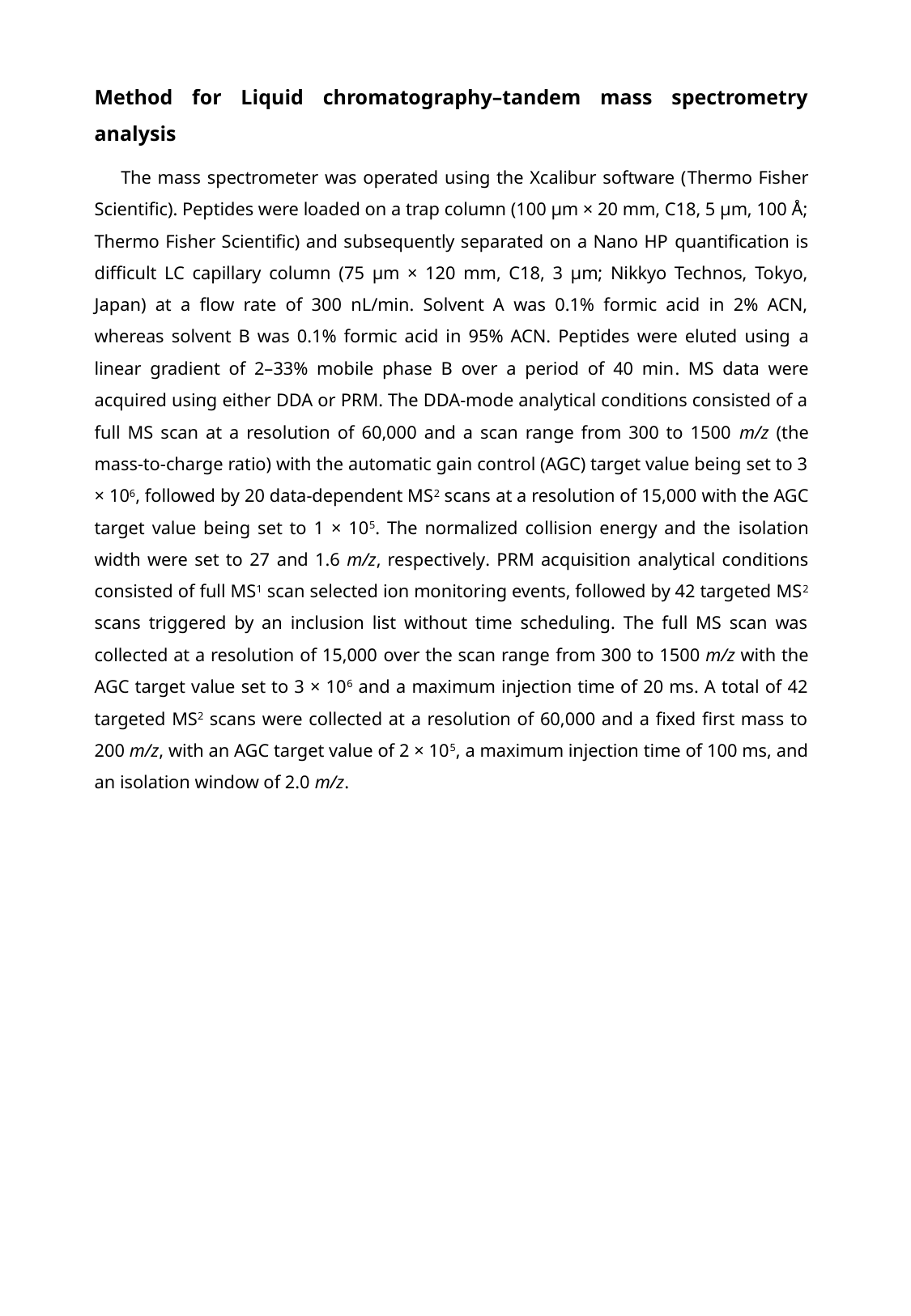

Method for Liquid chromatography–tandem mass spectrometry analysis
The mass spectrometer was operated using the Xcalibur software (Thermo Fisher Scientific). Peptides were loaded on a trap column (100 μm × 20 mm, C18, 5 μm, 100 Å; Thermo Fisher Scientific) and subsequently separated on a Nano HP quantification is difficult LC capillary column (75 μm × 120 mm, C18, 3 μm; Nikkyo Technos, Tokyo, Japan) at a flow rate of 300 nL/min. Solvent A was 0.1% formic acid in 2% ACN, whereas solvent B was 0.1% formic acid in 95% ACN. Peptides were eluted using a linear gradient of 2–33% mobile phase B over a period of 40 min. MS data were acquired using either DDA or PRM. The DDA-mode analytical conditions consisted of a full MS scan at a resolution of 60,000 and a scan range from 300 to 1500 m/z (the mass-to-charge ratio) with the automatic gain control (AGC) target value being set to 3 × 10^6^, followed by 20 data-dependent MS2 scans at a resolution of 15,000 with the AGC target value being set to 1 × 10^5^. The normalized collision energy and the isolation width were set to 27 and 1.6 m/z, respectively. PRM acquisition analytical conditions consisted of full MS1 scan selected ion monitoring events, followed by 42 targeted MS2 scans triggered by an inclusion list without time scheduling. The full MS scan was collected at a resolution of 15,000 over the scan range from 300 to 1500 m/z with the AGC target value set to 3 × 10^6^ and a maximum injection time of 20 ms. A total of 42 targeted MS2 scans were collected at a resolution of 60,000 and a fixed first mass to 200 m/z, with an AGC target value of 2 × 10^5^, a maximum injection time of 100 ms, and an isolation window of 2.0 m/z.

## Slide 3
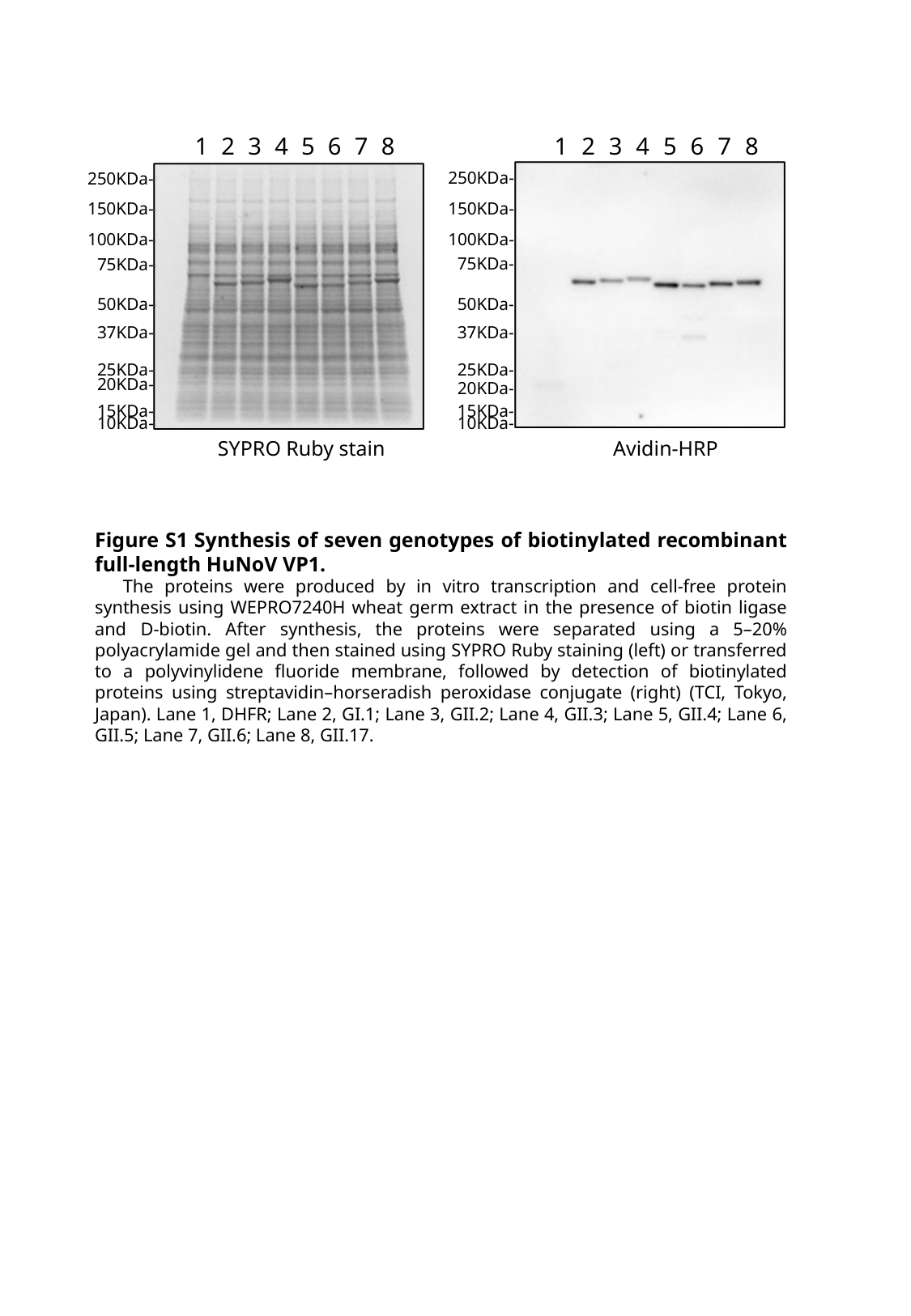

1 2 3 4 5 6 7 8
1 2 3 4 5 6 7 8
250KDa-
250KDa-
150KDa-
150KDa-
100KDa-
100KDa-
75KDa-
75KDa-
50KDa-
50KDa-
37KDa-
37KDa-
25KDa-
25KDa-
20KDa-
20KDa-
15KDa-
15KDa-
10KDa-
10KDa-
SYPRO Ruby stain
Avidin-HRP
Figure S1 Synthesis of seven genotypes of biotinylated recombinant full-length HuNoV VP1.
　The proteins were produced by in vitro transcription and cell-free protein synthesis using WEPRO7240H wheat germ extract in the presence of biotin ligase and d-biotin. After synthesis, the proteins were separated using a 5–20% polyacrylamide gel and then stained using SYPRO Ruby staining (left) or transferred to a polyvinylidene fluoride membrane, followed by detection of biotinylated proteins using streptavidin–horseradish peroxidase conjugate (right) (TCI, Tokyo, Japan). Lane 1, DHFR; Lane 2, GI.1; Lane 3, GII.2; Lane 4, GII.3; Lane 5, GII.4; Lane 6, GII.5; Lane 7, GII.6; Lane 8, GII.17.

## Slide 4
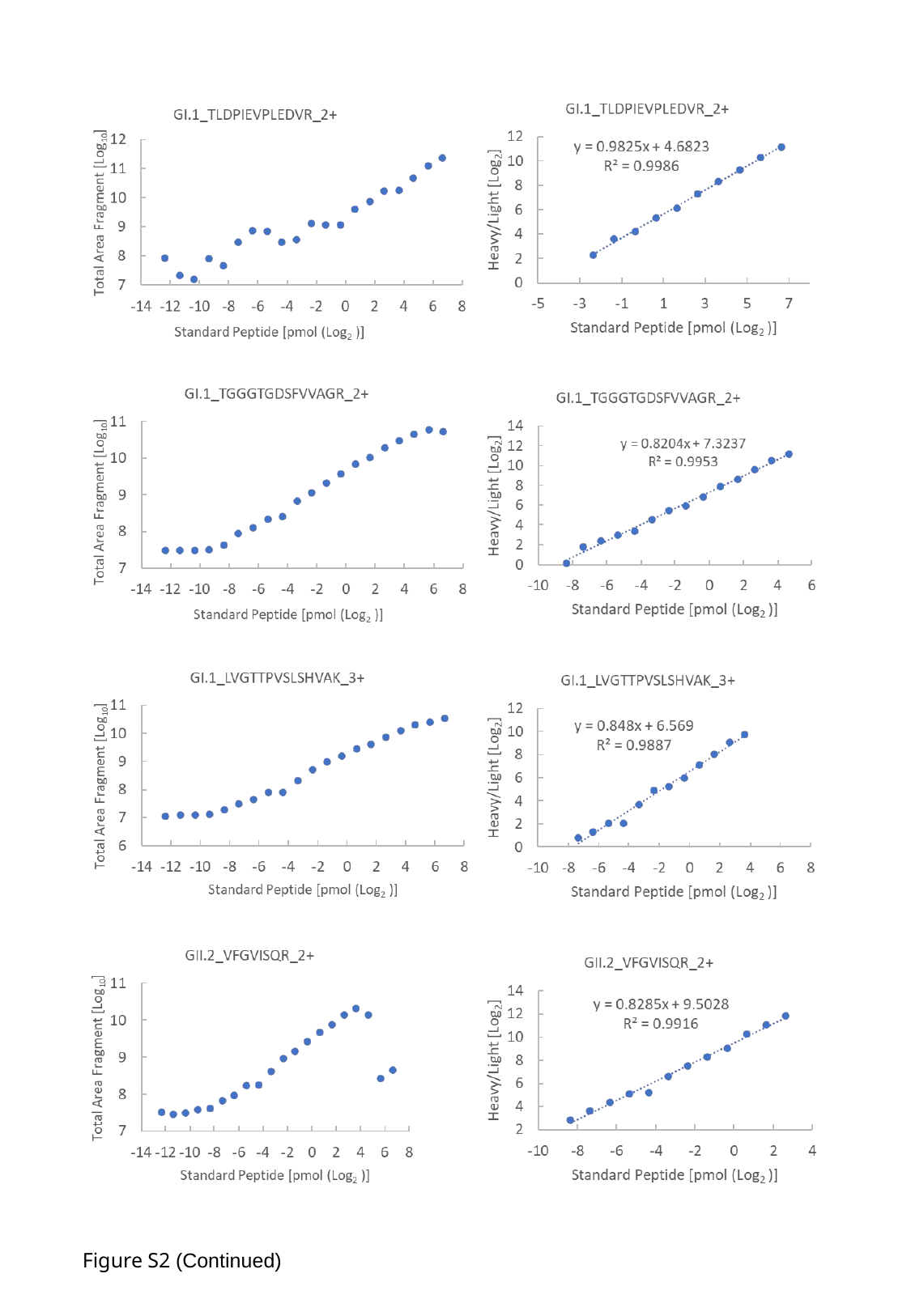

Figure S2 (Continued)

## Slide 5
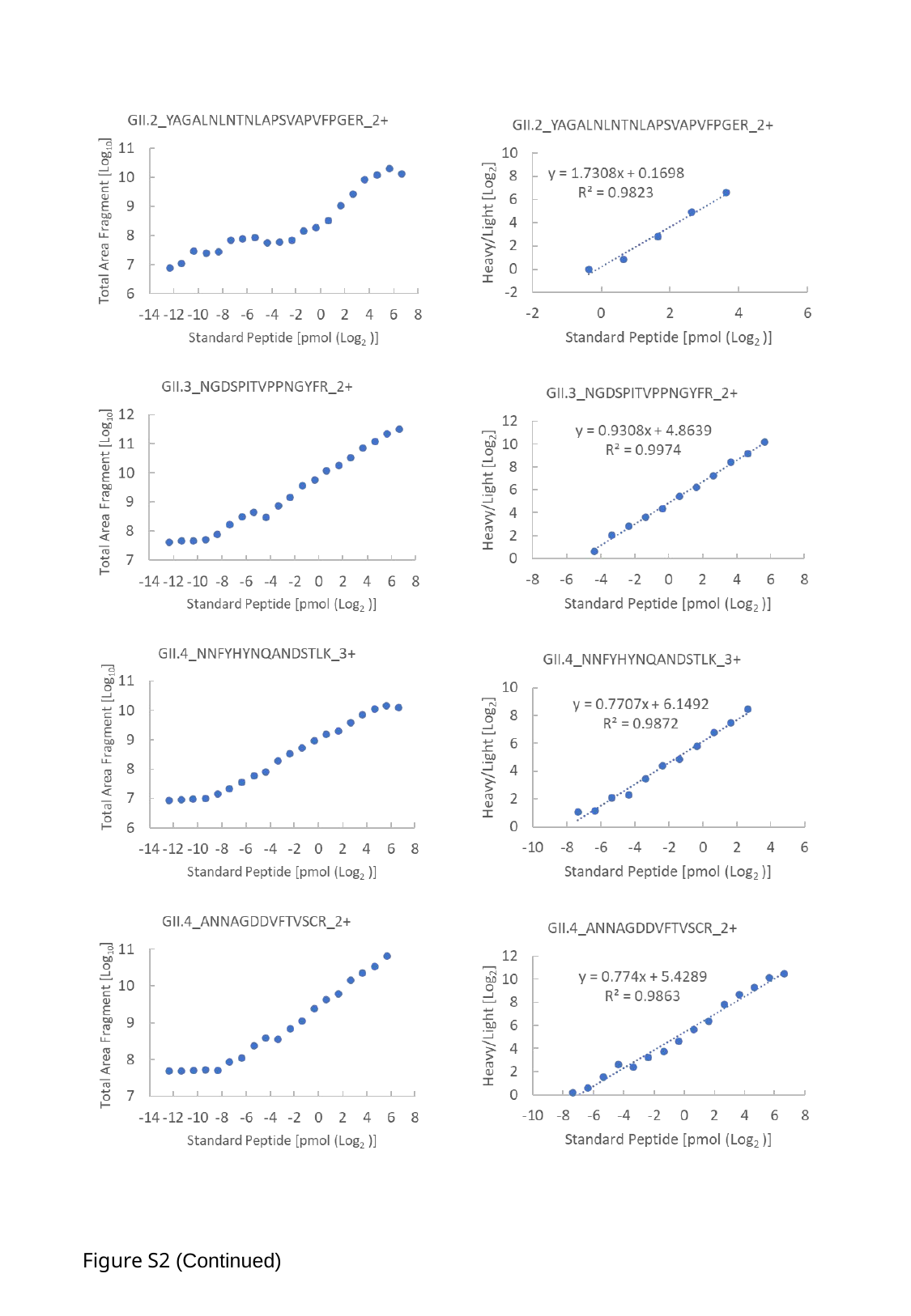

Figure S2 (Continued)

## Slide 6
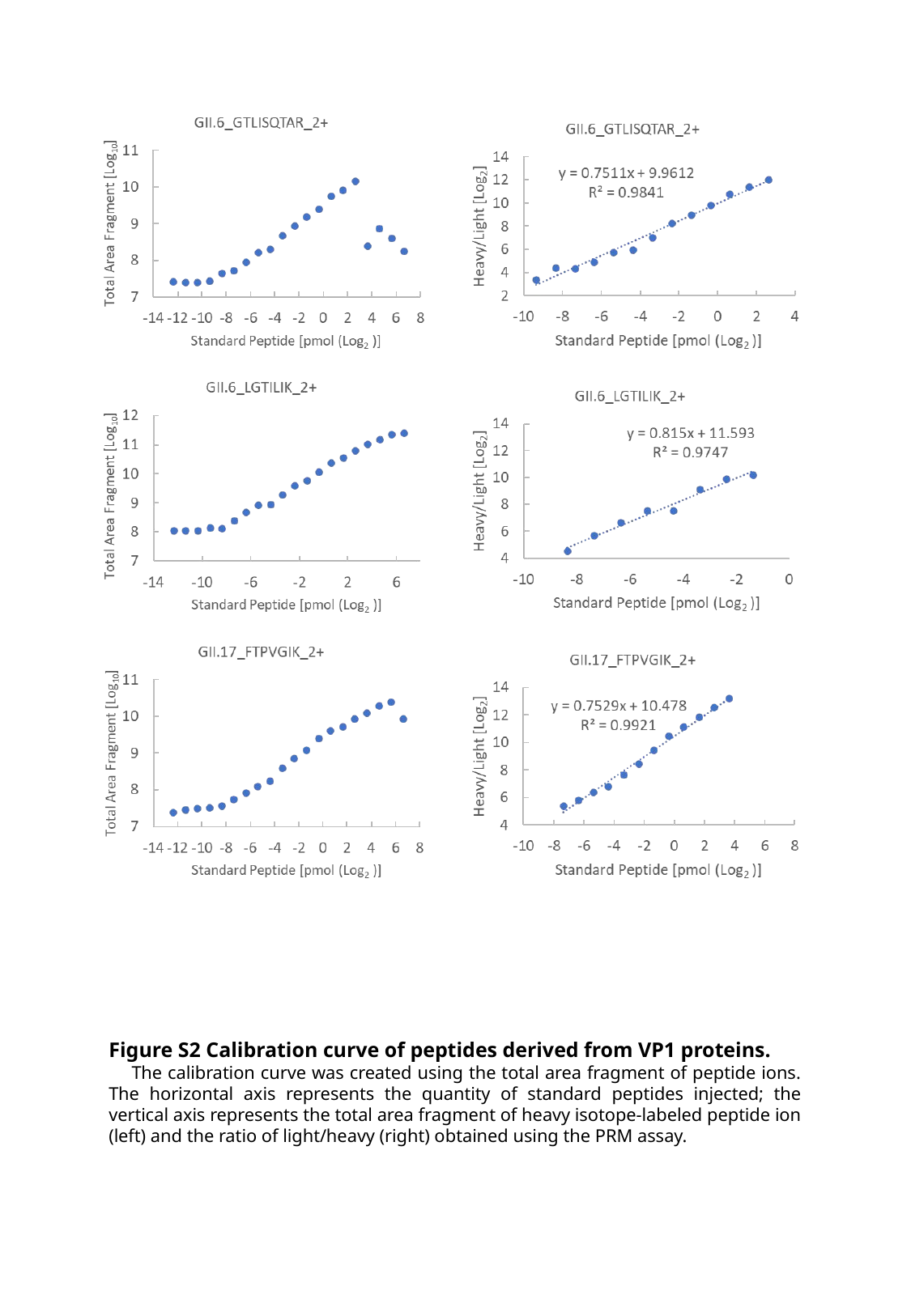

Figure S2 Calibration curve of peptides derived from VP1 proteins.
　The calibration curve was created using the total area fragment of peptide ions. The horizontal axis represents the quantity of standard peptides injected; the vertical axis represents the total area fragment of heavy isotope-labeled peptide ion (left) and the ratio of light/heavy (right) obtained using the PRM assay.
